# COVID-19 crisis: Influence of eHealth literacy on mental health promotion among Saudi nursing students

**DOI:** 10.1016/j.jtumed.2021.07.008

**Published:** 2021-08-11

**Authors:** Ejercito M. Balay-odao, Nahed Alquwez, Fatmah Alsolami, Hanan M.M. Tork, Khalaf Al Otaibi, Abdulellah Al Thobaity

**Affiliations:** a Nursing Department, College of Applied Medical Sciences, Shaqra University, Al Dawadmi, KSA; b Faculty of Nursing, Umm Al-Qura University, Makkah, KSA; c College of Nursing, Qassim University, Qassim, KSA; d College of Applied Medical Sciences, Taif University, Taif, KSA

**Keywords:** كوفيد-١٩, المعرفة الصحية الإلكترونية, تعزيز الصحة النفسية, الصحة النفسية الإيجابية, طلاب التمريض, السعودية, COVID-19, eHealth literacy, KSA, mental health promotion, nursing students, positive mental health

## Abstract

**Objective:**

This study aims to determine the influence of eHealth literacy on mental health promotion among Saudi nursing students.

**Method:**

This cross-sectional study was conducted from 19 April to 21 May 2020. A total of 468 nursing students responded to the self-administered online survey. Two instruments, namely, the eHealth Literacy Scale and the Positive Mental Health Scale, were used. Statistical tools such as frequency, percentage, mean, and standard deviation were used for the descriptive analysis. Multiple regression analysis was employed to analyse the relationship between eHealth literacy, health promotion, and its predictors.

**Results:**

The results showed that 30.3% of nursing students were dissatisfied with their general health. Students perceived high self-assessed eHealth literacy and positive mental health. Students in the 4th year had lower scores than those of students in the 2nd year. Nursing students perceived that the quarantine and public social distancing lowered their mental health scores. Finally, the eHealth literacy mean scores resulted in a 0.21-point increase in the positive mental health scores.

**Conclusion:**

The finding of the study shows that the eHealth literacy positively influnce the mental health status of Saudi Nursing students.

## الملخص

**أهداف البحث:**

هدفت هذه الدراسة إلى تحديد تأثير المعرفة الصحية الإلكترونية في تعزيز الصحة النفسية لدى طلاب التمريض السعوديين.

**طرق البحث:**

أجريت هذه الدراسة المقطعية في الفترة من ١٩ أبريل إلى ٢١ مايو ٢٠٢٠. أجاب ما مجموعه ٤٦٨ من طلاب التمريض على الاستبانة عبر الإنترنت. تم استخدام أداتين، وهما مقياس معرفة القراءة والكتابة في الصحة الإلكترونية ومقياس الصحة العقلية الإيجابي. تم استخدام التردد والنسبة المئوية والمتوسط والانحراف المعياري للتحليل الوصفي. كما استخدم تحليل الانحدار المتعدد لتحليل العلاقة بين المعرفة الصحية الإلكترونية، وتعزيز الصحة، والتنبؤات.

**النتائج:**

أظهرت النتائج أن ٣٠.٣٪ من طلاب التمريض غير راضين عن صحتهم العامة. ولاحظ الطلاب ارتفاع مستوى معرفة القراءة والكتابة في مجال الصحة الإلكترونية والصحة العقلية الإيجابية. حصل طلاب السنة الرابعة على درجات أقل من طلاب السنة الثانية. وأدرك طلاب التمريض أن الحجر الصحي والتباعد الاجتماعي العام يقللان من درجات الصحة العقلية الإيجابية. أخيرا، أدت درجة المعرفة في الصحة الإلكترونية إلى زيادة قدرها ٠.٢١ نقطة في درجات الصحة العقلية الإيجابية.

**الاستنتاجات:**

تقدم هذه الدراسة معلومات حول تأثير المعرفة الصحية الإلكترونية على الصحة النفسية الإيجابية لدى طلاب التمريض السعوديين.

## Introduction

Coronavirus disease 2019 (COVID-19) is a worldwide health problem that affects different sectors in society, particularly schools and universities that have remained closed since the disease was declared widespread and contagious. Mass quarantine is a common intervention imposed to contain and prevent the spread of the virus. The continuously increasing number of cases prolongs the quarantine period and gives rise to associated mental health problems due to loss of autonomy, ambiguity over disease situations, and boredom.^1^ Individuals who are under quarantine manifest psychological issues such as depression, mood disorders, and anxiety.^2^ According to a screening test conducted by Mental Health America,^3^ the occurrence of clinical anxiety increased during the first weeks of February and amplified by 12% during the first half of March 2020. Nursing students are not exempt from the resulting mental health problems. The sudden shift of classroom lectures to online platforms^4^ and the cancellation of clinical duties have been a concern for nursing students.^5^ Mental health problems such as stress and depression were some of the major concerns of nursing students even before the pandemic.^6^^,^^7^ In KSA, nursing students were found to have moderate psychological distress and moderate psychological wellbeing during the COVID-19 crisis.^8^

With the alarming rate of the emergence of mental health issues, mental health promotion has become essential to attain good health outcomes. A myriad of web pages provide information on mental health promotion in the wake of the ongoing COVID-19 pandemic and crises arising from it. The internet is the most accessible source of information for students of this generation and is utilised to answer their inquiries about their health.^9^ Accessing information on the internet could, however, also prove detrimental owing to a massive number of websites that provide fake or incomplete information.^10^ Therefore, many eHealth webpages were established to ensure the accuracy of health information^11^ and provide tips or information to the public regarding improving their mental health.^12^ Accessing this type of information requires a person to possess eHealth literacy; however, the determinants of the eHealth literacy of a student in promoting positive mental health are based on their demographic and psychographic data.^12^ In this context, Yang, Lou, and Chiang^13^ mentioned that individual factors could predict the eHealth literacy and health promotion behaviour of nursing students.

eHealth literacy is the skill of an individual to search, discover, recognise, and evaluate or appraise health information from the internet for health promotion.^14^ eHealth information is accessible using mobile phones and computers but requires the person to be literate and digitally aware.^15^ Such webpages are not new to nursing students because they are required to read and search articles on reliable medical webpages or access evidence-based databases.^16^ Furthermore, nursing students have advanced eHealth literacy because they frequently seek health information and are generally highly concerned about their health.^17^

In KSA, the use of eHealth information is growing. Many institutions, groups, and individuals have implemented the application of eHealth; thus, research studies are available in the literature about eHealth.^18^, ^19^, ^20^ However, the existing studies do not reflect the current application of eHealth in the country since the data available are only collected from a few organisations.^21^ With the growing available literature on eHealth literacy, few research studies have shown the relationship between eHealth literacy and psychological wellbeing.^13^^,^^17^ In the kingdom, no study conducted determined the influence of eHealth literacy on the psychological wellbeing of nursing students. Although, some studies did describe the impact of eHealth literacy on the health promotion behaviours of medical students.^22^, ^23^, ^24^ This gap in knowledge prompted the researchers to conduct this study knowing that studies have revealed that the effect of eHealth literacy on psychological wellbeing may vary based on the culture, country, and levels of internet usage.^13^ According to Chiang, Yang, and Hsu,^12^ the adopting behaviour of a student is based on the level of his/her eHealth literacy. It was observed that most students in the medical field have high eHealth literacy and engage in optimistic health promotion behaviour.^17^ However, a study in Jordan revealed that nursing students could not categorise internet information in order of accuracy.^25^ Data uncertainty makes it difficult to ensure that the accessed information is an evidenced-based intervention. Hence, eHealth literacy is crucial for helping individuals to access information on mental health promotion activities. An example constitutes nursing students who can act as mental health promoters capable of addressing their psychological issues and those of family members, patients, and the society. Therefore, understanding the relationship between eHealth literacy and the promotion of positive mental health of nursing students is essential.

## Objective of the study

This study aimed to determine the influence of eHealth literacy mental health promotion among Saudi nursing students.

### Materials and Methods

A cross-sectional design was used to obtain the relationship among individual factors, eHealth literacy, and positive mental health of nursing students. This study was conducted using an online self-administered questionnaire that was distributed to the personal Facebook and Twitter accounts of nursing students, which have been utilised by the student for any important messages.

### Sample

The respondents were nursing students from four state universities in KSA. University one (1) is situated in the Riyadh region, University two (2) is located in the Al-Qassim region, and Universities three (3) and four (4) are located in the Makkah region. The availability of collaborators in these universities prompted the researchers to consider the locale of the study. A sample size of 468 nursing students participated from 19 April to 21 May 2020. Inclusion criteria were as follows: the participants were enrolled nursing students in the participating university during data gathering irrespective of year level, were of any gender, had personal Facebook and Twitter accounts, and were willing to participate in the study.

### Survey instruments

Two instruments, namely, the eHealth Literacy Scale (eHEALS) and Positive Mental Health Scale (PMHS), were used with permission granted by the authors. In addition, the demographic profile of the respondents was obtained.

eHEALS^12^ is an eight-item questionnaire used to measure the eHealth literacy of nursing students. eHealth literacy was developed to evaluate the information, comfort, and perceived skills of a person at discovering, appraising, and utilising learned electronic health information in promoting wellbeing.^12^ Each question is measured on a scale of 1–5 interpreted as strongly disagree to strongly agree. A high eHEALS score indicates high self-assessed eHealth literacy. This tool has good test-retest reliability with .89 to .97 internal consistency reliability.^12^ Two supplemental questions are indicated by the eHEALS authors to assess the importance of accessing health resources and measure the usefulness of the internet for making health decisions.

PMHS is a short nine-item, one–dimensional scale used to measure positive mental health. The PMHS-scale is used for a quick overall assessment of positive mental health in the community and mental health facilities. Each item is rated on a four-point Likert scale ranging from 1 to 4 interpreted as do not agree to agree. A high score for positive mental health represents high self-assessed positive mental health. Internal consistency on the reliability of alpha of .93 and test-retest methods revealed the good reliability of this tool.^26^

### Data gathering

An online survey was conducted by sending a personal message to participants or posting invitations in the personal social media account of students such as Twitter and Facebook. This invitation contains the consent form informing them of their rights and responsibility and that no identifying data that could link them to their responses would be included. If the respondents are interested, then they click on the embedded web link provided to access the questionnaires. The web link consists of the welcome screen inviting the nursing students to participate and the consent form. An instruction on the web link asking the willing nursing students to carefully read the consent form before clicking the “I accept” was also indicated. After the student consented to the participant, the demographic of the student will appear, and then followed by the two questionnaires and a statement thanking the student for their participation. Once the student is done answering the questions, he/she will click the submit icon. The questionnaire took approximately 10–15 minutes. The weblink was closed after data gathering, and the data were extracted and stored in office files on a password-protected computer. Access to data was provided only for the researchers and other institutions with legal permission.

### Ethical consideration

This study sought the approval of the Institution Research Board of Shaqra University Al-Dawadmi, KSA (RU-0023). The informed consent was taken from the participants that states the research objective and aim, voluntary participation, right to autonomy and confidentiality, and the right to withdraw to participate in the study. In addition, the web link did not gather IP addresses or any supplementary data.

### Statistical analysis

Data were analysed using SPSS software. Frequency, percentage, mean, and standard deviation were used for descriptive analysis. Multiple regression analysis was employed to analyse the relationship among eHealth literacy, health promotion, and its predictors.

## Results

468 samples were included. As reflected in Table 1, the proportion of sample size from lowest was university 3, 1, 2, and 4, with the percentage, are 7.1%, 27.4%, 27.8%, and 37.8%, respectively. The recorded samples were from 3rd, 2nd, 4th, and internship year nursing students, with a percentage of 43.6%, 35.5%, 16.9%, and 4.1%, respectively. The majority of the respondents were female (73.9%) and belonged to a nuclear type of family (79.7%). The mean age was 21.44 years (SD = 1.73).

**Table 1:** Demographic variables of the respondents (N = 468).

Variable	n	%
University		
University 1	128	27.4
University 2	130	27.8
University 3	33	7.1
University 4	177	37.8
Gender		
Female	346	73.9
Male	122	26.1
Year level		
2nd year	166	35.5
3rd year	204	43.6
4th year	79	16.9
Internship year	19	4.1
Family structure		
Nuclear	373	79.7
Extended	95	20.3
Perceived mental health status		
Bad	15	3.2
Moderate	178	38.0
Good	275	58.8
Perceived general health status		
Very dissatisfied	4	0.9
Dissatisfied	142	30.3
Neutral	61	13.0
Satisfied	139	29.7
Very satisfied	122	26.1
	Mean	SD
Age	21.44	1.73
Perceived effect of quarantine on mental health	3.89	2.67
Perceived effect of social distancing on mental health	3.93	2.80
Perceived effect of mass gathering cancellation on mental health	3.67	2.72
Perceived effect of university closure on mental health	4.16	2.96

In terms of psychographic, the majority of the respondents reported good mental health status (58.8%), and only 3.2% reported having a bad mental health status. There are high percentages of the respondents who perceived their general health status as “dissatisfied (30.3%). The respondents’ mean scores in their self-assessed perceived effects of quarantine on mental health, public social distancing on mental health, mass gathering cancellation on mental health, and university closure on mental health were 3.89 (SD = 2.67), 3.93 (SD = 2.80), 3.67 (SD = 2.72), and 4.16 (SD = 2.96), respectively (see Table 1).

The results of descriptive analyses on the students’ eHealth literacy are summarised in Table 2. Most students exhibited a high level of self-assessed eHealth literacy, as evidenced by the mean score of 3.76. Almost all nursing students perceived the highest mean for the statement “How useful do you feel the internet is in helping you in making decisions about your health?” (4.05). The statements that received the highest mean scores were “How important is it for you to be able to access health resources on the internet?” (3.94), the statement, “Nursing students know how to use the internet to answer their questions about health” had a mean of 3.88, followed by the statements, “I know how to use the health information I find on the internet to help me” (3.88), and “I have the skills I need to evaluate the health resources I find on the internet” (3.76). The statements with the lowest mean score were “I feel confident in using information from the internet to make health decisions” (3.59) and “I know what health resources are available on the internet” (3.52).

**Table 2:** Results of the descriptive analysis on the eHealth Literacy Scale (N = 468).

Variable	Mean	SD
How useful do you feel the internet is in helping you in making decisions about your health?	4.05	0.76
How important is it for you to be able to access health resources on the internet?	3.94	0.83
I know what health resources are available on the internet	3.52	0.96
I know where to find helpful health resources on the internet	3.60	0.90
I know how to find helpful health resources on the internet	3.71	0.83
I know how to use the internet to answer my questions about health	3.88	0.74
I know how to use the health information I find on the internet to help me	3.87	0.74
I have the skills I need to evaluate the health resources I find on the internet	3.76	0.78
I can tell high-quality health resources from low-quality health resources on the internet	3.68	0.79
I feel confident in using information from the internet to make health decisions	3.59	0.88
Overall mean	3.76	0.56

Table 3 presents the result of the descriptive analyses on the positive mental health of nursing students. The nursing students perceived high self-assessed positive mental health (3.31). The highest mean score was for the statement “I manage well to fulfil my needs” (3.40), followed by “I am a calm, balanced human being” (3.37). The statement “I feel that I am well equipped to deal with life and its difficulties” received a mean score of 3.35, and “I am in good physical and emotional condition” had a mean score of 3.34. Most of the nursing students least agreed on the statement “I enjoy my life” (3.25) and “I am often carefree and in good spirits” (3.21).

**Table 3:** Results of the descriptive analyses on the Positive Mental Health Scale (N = 468).

Variable	Mean	SD
I am often carefree and in good spirits.	3.21	0.65
I enjoy my life.	3.25	0.66
All in all, I am satisfied with my life.	3.28	0.68
In general, I am confident.	3.32	0.61
I manage well to fulfil my needs.	3.40	0.57
I am in good physical and emotional condition.	3.34	0.65
I feel that I am well equipped to deal with life and its difficulties.	3.35	0.63
Much of what I do brings me joy.	3.27	0.66
I am a calm, balanced human being.	3.37	0.65
Overall mean	3.31	0.46

Standard multiple regression analysis was conducted to examine the predictors of the students' positive mental health. The regression model was statistically significant (*F*[16, 451] = 6.44, *p* < .001) and accounted for 15.7% of the variance in positive mental health (*R*^*2*^ = 0.168; Adjusted *R*^*2*^ = 0.157). As shown in Table 4, the students' year level, perceived effect of quarantine on mental health, perceived effect of public social distancing on mental health, and eHealth literacy were significant predictors of their positive mental health. The 4th year students had lower scores by 0.16 (*p =* .011, 95% CI = −0.28, −0.04) than the 2nd year students. A point increase in the students’ perceived effect of quarantine on mental health corresponded to a decrease of 0.03 points (*p* = .009, 95% CI = −0.05, −0.01) in the positive mental health score. Similarly, a point increase in the perceived effects of public social distancing on mental health resulted in a decrease of 0.04 points (*p* = .001, 95% CI = −0.06, −0.02) in the positive mental health score. Finally, a point increase in the eHEALS mean score resulted to an increase of 0.21 point (*p* < .001, 95% CI = 0.14, 0.28) in the positive mental health scores.

**Table 4:** Factor influencing positive mental health (N = 468).

Predictor variable	β	SE-*b*	Beta	*t*	*p*	95% CI	
						Lower	Upper
University (Reference group: University 4)							
University 1	0.05	0.06	0.05	0.92	.358	−0.06	0.17
University 2	0.06	0.06	0.06	0.95	.343	−0.06	0.18
University 3	0.04	0.10	0.02	0.48	.632	−0.14	0.22
Gender	0.07	0.06	0.07	1.24	.216	−0.04	0.18
Year level (Reference group: 2nd year)							
3rd year	−0.08	0.05	−0.08	−1.50	.135	-.018	0.02
4th year	−0.16	0.06	−0.13	−2.55	.011∗	−0.28	−0.04
Internship year	0.06	0.12	0.02	0.47	.641	−0.18	0.30
Age	0.00	0.01	0.01	0.11	.913	−0.02	0.03
Family structure	0.09	0.05	0.08	1.72	.085	−0.01	0.19
Perceived mental health status	0.03	0.04	0.04	0.75	.456	−0.05	0.10
Perceived general health status	0.02	0.02	0.06	1.21	.228	−0.01	0.06
Perceived effect of quarantine on mental health	−0.03	0.01	−0.15	−2.62	.009∗∗	−0.05	−0.01
Perceived effect of social distancing on mental health	−0.04	0.01	−0.25	−3.40	.001∗∗	−0.06	−0.02
Perceived effect of mass gathering cancellation on mental health	0.02	0.01	0.13	1.83	.068	−0.00	0.04
Perceived effect of university closure on mental health	−0.00	0.01	−0.02	−0.39	.696	−0.02	0.01
Overall mean E-Heals	0.21	0.04	0.26	5.93	<.001∗∗∗	0.14	0.28

Note. Positive mental health is the dependent variable. β is the unstandardised coefficients; SE-b is the Standard error. Beta is the standardised coefficients.

*R*^*2*^ = 0.168; Adjusted *R*^*2*^ = 0.157.

∗Significant at 0.05, ∗∗Significant at .01, ∗∗∗Significant at 0.001.

## Discussion

This study examined the variables influencing positive mental health. The percentage of nursing students who are dissatisfied with their mental health is high (30.3%). This finding could be associated with the COVID-19 pandemic that affects the learning of students and their mental health. This present study concurs with the study conducted in Lebanon, wherein the authors revealed that during the pandemic, student learning was compromised and caused stress due to loads of requirements.^27^ Additionally, a qualitative study conducted in KSA revealed that students experience multiple challenges such as problems with understanding the topic being presented by the instructor, technical and behavioural problems during online lectures, and online examinations.^17^ Thus, the student's mental state must be considered during the crisis to ensure quality and appropriate psychological support.^28^

Furthermore, this study found that nursing students in KSA have high eHealth literacy. Similarly, a study in Japan,^29^ Iran,^30^ and America^31^ reported that nursing students have high eHealth literacy. This finding of the study could be associated with the awareness of the Saudi people on how to access the internet since KSA is one of the highest numbers of people using the internet at 74.8% of the population as of 2018.^9^ The finding could also be associated with the nature of the course requirement of the nursing students. Their subjects require them to access the internet for health information and to enhance their learning further. The findings of the study also showed that the statement “How useful do you feel the internet is in helping you in making decisions about your health?” has a high mean score. This finding could be associated with the use of social media by the health authorities to convey COVID-19 information. In KSA, the Ministry of Health (MOH) used Facebook, Twitter, and other social media platform to inform the public about the disease, thereby further enhancing the knowledge and skill of students' nurses on deciding the best site to look for credible information about their mental health.^32^ In addition, students used the internet to access or obtain information about their health, and contributed to the usefulness and importance of this tool.^33^ Today's generation places high importance on information technology in their lives^10^ and uses this platform to answer their queries and information about their health. However, the vast amount of information found on the internet sometimes leads to confusion among nursing students, making them poorly confident to utilise the information.^13^

As to the positive mental health of the nursing student during this pandemic, they perceived that they could manage to fulfil their needs. This finding is associated with the family structure of the nursing student, the study does not show a significant difference in the influence of the family structure, but the family function might have an impact on the positive mental health of the nursing student. Most of the nursing students belong to the nuclear family structure, where there is more cohesion and communication.^34^ Family cohesion has been identified as a factor that promotes positive mental health.^35^ Furthermore, for Muslims, the family unit is considered important to maintain a healthy and balanced society.^36^ A healthy family relationship makes nursing students satisfied and happy. This relationship also contributes to why nursing students are calm, balanced human beings that are equipped to deal with life difficulties. Thomas, Liu, and Umberson^37^ mentioned that a strong family relationship positively influences the mental health of a person. A healthy family relationship helps nursing students cope with any challenges, especially during this COVID-19 pandemic. In addition, nursing students are taught how to deal with stressful situations. Their increased knowledge of mental health promotion helps them maintain stable physical and emotional states.^13^ However, this pandemic slightly affects the feeling of enjoyment, carefree attitude, and good spirit of nursing students.^29^^,^^38^

Furthermore, quarantine and public social distancing were found that influence the positive mental health of nursing students. Although quarantine and public social distancing are well-established preventive measures of COVID-19,^14^ these practices negatively affect the mental health of nursing students.^1^ Brook et al.^1^ reported that quarantine increases the incidence of boredom, frustration, anxiety, depression, and confusion. Similarly, public social distancing contributes to the increase in psychological problems such as anxiety and depression.^39^ These factors also negatively affect nursing education, and therefore, the mental suffering of nursing students.^40^ Thus, this required a measure to reduce student mental pain and suffering to ensure a positive learning attitude and.^41^

The year level of nursing students influenced their positive mental health. It was noted in the study that 2nd year students perceived positive mental health as compared with 4th year nursing students. This finding could be associated with the pressure being experienced by nursing students concerning their studies. The 2nd year nursing students are focused on the fundamentals of nursing, where basic information on nursing concepts is being taught as compared to 4th year nursing students whose focus is on medical disorders and management. This nature of learning in nursing has identified a cause of stress and mental health issues.^42^^,^^43^ Chernomas and Shapiro^44^ mentioned that the causes of mental issues of nursing students are related to too much course load, their aim to have a high general mark average, stringent examinations, colleges in the clinical duties and nursing laboratories, and complex interpersonal relationships.

The result of the study also supported that eHealth literacy influences the positive mental health of nursing students. Nursing students are greatly concerned about their mental health, leading them to frequently use web-based health information and consequently increasing their mental health promotion.^13^^,^^42^^,^^44^ The usefulness and importance of web-based health information for nursing students also help to promote their positive mental health.^31^ Most eHealth services are interactive, making the learning informative and interesting and the information easily transferrable and applicable to a person^45^ in a specific situation. The high eHealth literacy of nursing students indicates their ability to appraise information on the internet and hone their decision-making skills on utilising information for specific situations.^30^ Therefore, nursing students can discern an intervention that they utilised in the promotion of their mental health.^31^ This finding also further show that the health promotion activities derived from eHealth source.^13^ As mentioned by Chen, Liang, and Tsai,^46^ health information obtained on the internet affects a person's decision-making. This finding of the study implies the importance of the development of eHealth literacy since it helps in health promotion. Promoting student mental health is a significant consideration to remember to facilitate and promote their health status; thus, it enables the successful development of their nursing school programme with the progression to professional practice.

## Limitation of the study

This study did not assess the mental health status of the participants. The analysis would have been highly robust if this variable was included while controlling the predictors of eHealth and positive mental health. Moreover, this study found that quarantine and social distancing are predictors of poor mental health. Other confounding or mediating variables, such as the self-efficacy and resilience of nursing students, might have affected the results. Future works should include this factor to understand eHealth and positive mental health further.

## Conclusion

This study found that Saudi Arabian nursing students have high eHealth literacy and positive mental health. The 2nd year students have higher positive mental health than 4th year nursing students. The positive mental health of nursing students during the COVID-19 crisis was affected by the quarantine and public social distancing measures of the government. This study showed that nursing students with high eHealth literacy had positive mental health during the COVID-19 crisis.

## Recommendation

Based on the findings of the study, the researchers recommend the following: Faculty members, school administration, and government authorities in education should design or develop evidence-based activities and programmes to be implemented in the universities to enhance the eHealth literacy and positive mental health of nursing students. The finding of the study also signifies the need to strengthen the lecture on how nursing students appraise information and web pages for their mental health promotion. This method will help nursing students enhance their awareness of the appropriate webpages to enable access to mental health information and promote their mental health. In addition, nursing students should be encouraged to develop their basic level of literacy to the critical thinking level of literacy as they progress in their year level. Interventions to strengthen the importance of mental health must be considered in the activities and programmes for nursing students. Nursing education facilitators should aim to further enhance and develop the competencies of their students regarding their eHealth literacy to ensure that their positive mental health is maintained or ensured. Further qualitative study is recommended to understand and confirm the different perspectives of these year levels regarding the influence of eHealth literacy on positive mental health among nursing students.

## Source of funding

The authors did not receive any specific grant from funding agencies in the public, commercial, or not-for-profit sectors.

## Conflict of interest

The authors have no conflict of interest to declare.

## Ethical approval

This study was approved by **The University Research Committee with approval number (RU-0023)** and ensured the ethical conduct of the study issued 10 April 2020.

## Consent

Informed consent was obtained from the participants after explaining to them the research objective and aim, voluntary participation, right to autonomy and confidentiality, and the right to withdraw from participation in the study.

## Authors contributions

EMB- conceived and designed the study, conducted research, provided research materials, and collected and organised data. EMB-CD, and NA analysed and interpreted the data.

FA, HT, KAO, AAT provided logistic support and collected data. All authors were involved in writing the initial and final draft of the article. All authors have critically reviewed and approved the final draft and are responsible for the content and similarity index of the manuscript.
